# Contribution to the Improvement of the Correlation Filter Method for Modal Analysis with a Spatial Light Modulator

**DOI:** 10.3390/mi13112004

**Published:** 2022-11-17

**Authors:** David Benedicto, María Victoria Collados, Juan C. Martín, Jesús Atencia, Omel Mendoza-Yero, Juan A. Vallés

**Affiliations:** 1 Departamento de Física Aplicada, Instituto de Investigación en Ingeniería de Aragón (I3A), Facultad de Ciencias, Universidad de Zaragoza, Pedro Cerbuna 12, 50009 Zaragoza, Spain; 2 Institut de Noves Tecnologies de la Imatge (INIT), Universitat Jaume I, 12080 Castelló, Spain

**Keywords:** modal decomposition, correlation filter method, spatial light modulator, double phase method, multimode fibers, computer generated hologram, modal division multiplexing

## Abstract

Modal decomposition of light is essential to study its propagation properties in waveguides and photonic devices. Modal analysis can be carried out by implementing a computer-generated hologram acting as a match filter in a spatial light modulator. In this work, a series of aspects to be taken into account in order to get the most out of this method are presented, aiming to provide useful operational procedures. First of all, a method for filter size adjustment based on the standard fiber LP-mode symmetry is presented. The influence of the mode normalization in the complex amplitude encoding-inherent noise is then investigated. Finally, a robust method to measure the phase difference between modes is proposed. These procedures are tested by wavefront reconstruction in a conventional few-mode fiber.

## 1. Introduction

Characterization of optical fields by means of modal decomposition (MD) is key for the analysis, design, and optimization of multimode waveguide-based devices [1,2,3]. Interest in these techniques is increasing in parallel with the growing research on applications based on multimode structures such as high-power large-mode-area fiber lasers [4,5] and space division multiplexing techniques, aimed at widening the channel capacity of optical communication systems [6,7,8]. Modal decomposition facilitates the study of modal competition [9,10], modal instabilities limiting the maximum power emission in fiber lasers [11,12], nonlinear optical effects where the interaction of many transversal modes is involved [13,14,15], and the design of optical devices based on optical power transfer [16,17] and selective mode excitation [18,19]. This analysis has proven to be a key tool to study the transmission properties of waveguides regarding its modal behavior due to the physical insight that can be obtained [20].

In general, MD methods can be classified as numerical or experimental. On one hand, several numerical MD algorithms based on measured optical intensity distributions have been reported [21]. Iterative methods such as the GS algorithm [22], the SPGD algorithm [23], and the genetic algorithm [24] show a high accuracy but are highly sensitive to the initial values due to the local minima problem and, in some cases, the necessary iterative process can be computationally intensive [25]. The newly emerged neural network methods [26,27,28] outperform the iterative methods in decomposition speed without having the local minima problems and showing a highly accurate performance, but require high-performance computers, a large amount of memory, and a long time for training the neural network. Recently, a non-iterative method based on a sophisticated analytical model has been published [29], achieving a very fast MD performance and without any optimization or training process, as long as the signal-to-noise ratio of the acquired images remains particularly low.

On the other hand, MD can be directly performed through experiments, such as by the use of ring-resonators [30], the evaluation of multimode interference commonly known as S^2^ [31] (spatially and spectrally resolved imaging) and C^2^ [32] (frequency domain cross-correlated imaging), low-coherence interferometry [33], the application of fiber Bragg gratings [34] or diffraction gratings [35], and the correlation filter method (CFM) [36]. The latter allows us to retrieve the full optical field information by measuring only a few modal amplitudes and phases. Compared to numerical methods, this procedure requires a more complex experimental setup, but it provides a fast and accurate MD without the need for developing any software for the analysis of the intensity distribution images.

In the CFM, the waveguide output beam illuminates a computer-generated hologram (CGH), acting as a match filter, which performs the decomposition of the output field into the waveguide propagation modes by means of a specific previously designed transmittance function. Hence, a priori knowledge of the field distribution of the waveguide-under-study set of modes is required. Therefore, the designed CGH would be limited to analyzing only the corresponding type of waveguide. This limitation can be overcome by implementing the CGH into a spatial light modulator (SLM), allowing the user to adapt the decomposition mode set so that any waveguide can be investigated [37].

Thanks to the recently emerged liquid-crystal SLM technology, the experimental procedure has been significantly simplified, and the CFM has been used for mode analysis [36,37,38], mode-resolved bend loss analysis [39], mode-resolved gain analysis [10], wavefront reconstruction [40], and mode excitation [20]. However, there are only a few works describing the details of this method [41], which could be related both to the novelty of the technology and to the complexity of the method compared to other MD approaches. Taking this into account, we provide in this work a series of operational procedures, not detailed to date in any paper as far as we know, in order to carry out this method correctly and to get the most out of it. This way, part of its complexity becomes reduced.

Correlation filters require light amplitude and phase modulation. Nevertheless, commercial SLMs can only induce a phase shift or an amplitude modulation. Both phase and amplitude modulation can be achieved with a phase-only SLM by conveniently grouping the SLMs pixels into what have been called macropixels [42,43,44,45,46], thus allowing the correlation filter method implementation. However, this technique gives rise to an output field presenting a noise term [45], which not only depends on the filter but also on the input-field distribution. In this work, we study the effect of the mentioned noise term, inherent to the use of macropixels when an SLM is employed in an MD setup.

While implementing the CFM, we have studied some factors that can condition and worsen its performance (e.g., system alignment, CGH adjustment, a priori mode computation, laser instabilities), for which we offer a set of techniques that can be useful in order to lessen its influence. On the one hand, we propose a CGH-scale-adjustment technique based on the waveguide mode symmetry in order to easily adjust the match-filter size. On the other hand, a different way of measuring the phase difference between modes is presented, reducing the system instabilities effects and improving the MD performance.

The manuscript is organized as follows. For the sake of completeness, Section 2 is devoted to revise the MD technique based on the CFM implemented in a phase-only SLM. In Section 3, the experimental setup is explained. Section 4 contains the mentioned noise-term analysis, inherent to the necessary encoding technique, while Section 5 and Section 6 include the proposed scale adjustment and phase-retrieval techniques, respectively. Finally, in Section 7 we test the experimental performance of the modal-analysis procedure by a wavefront reconstruction, which leads to the conclusions in Section 8.

## 2. The Correlation Filter Method for Modal Decomposition

### 2.1. Basics of the Correlation Filter Method

**Figure 1:**
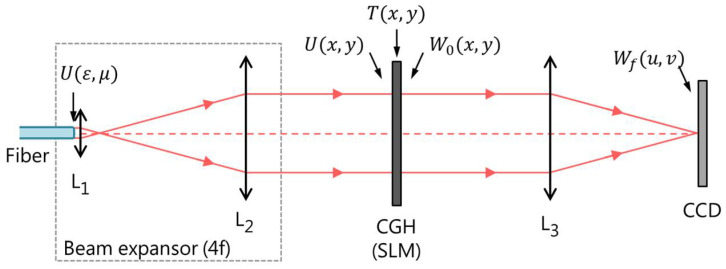
Scheme of the modal decomposition setup. L_1_, L_2_, and L_3_ are lenses. SLM is the spatial light modulator where the CGH is implemented. U(ε,μ) is the waveguide output electric field, U(x,y) is the electric field at the CGH plane after passing through the beam expansor in a 4f configuration, T(x,y) is the SLM transmittance, $W_{0}\left(x,y\right)$ is the electric field after the SLM, and $W_{f}\left(x,y\right)$ is the electric field at the CCD camera plane.

The notation to be employed is established in Figure 1, which shows a basic scheme of the setup necessary for modal decomposition using the CFM. Lenses L_1_ and L_2_, positioned following a 4f configuration and act as a beam expansor, where the fiber output end is placed at the focus of lens L_1_; the distance between lenses is the sum of their focal distances and the CGH is located at the focal plane of lens L_2_. Through this beam expansor, the fiber output electric field U(ε,μ) is imaged at the CGH plane, U(x,y). T(x,y) is the CGH transmittance implemented in the SLM, $W_{0}\left(x,y\right)$ is the electric field after the SLM, and $W_{f}\left(x,y\right)$ is the field at the CCD camera position, which is placed at the focal plane of lens L_3_.

The multimode waveguide output field to be analyzed constitutes the input CGH field

(1) $$U\left(x,y\right)=\overset{N}{\underset{n=1}{\sum }}c_{n}\phi_{n}\left(x,y\right).$$

where x and y are orthogonal coordinates in the CGH plane, N is the total number of propagation modes allowed by the waveguide under study [47], $\phi_{n}\left(x,y\right)$ is the nth-mode normalized distribution of the transversal electric field, and $c_{n}$ is its complex expansion coefficient, which can be expressed in terms of a modal weight, $|c_{n}|$, and a relative phase term, $\varphi_{n}$,

(2) $$c_{n}=|c_{n}|e^{{i\varphi }_{n}},$$

where i is the imaginary unit and where all mode relative phases are expressed with respect to the 0-mode phase $\left(\varphi_{0}=0\right)$.

The electric fields $W_{0}\left(x,y\right)$ and U(x,y) are related by:

(3) $$W_{0}\left(x,y\right)=U\left(x,y\right)T\left(x,y\right).$$

The Fourier transform of the output distribution, $W_{f}\left(u,v\right)$, is obtained at the L_3_ lens focal plane. Specifically, $W_{f}$ and $W_{0}$ are related by means of [48]:

(4) $$W_{f}\left(u,v\right)=\frac{1}{i\lambda f}ℱ\left[W_{0}\left(x,y\right)\right],$$

where ℱ denotes Fourier transform, λ is the input-light wavelength, f is the lens focal distance, and the coordinates at the focal plane (u,v) are related to the Fourier-transform frequency-space coordinates $f_{x}$ and $f_{y}$ by means of ${u=\lambda \mathrm{ff}}_{x}$, ${v=\lambda \mathrm{ff}}_{y}$. In order to measure the amplitude of mode p, a filter with the following transmittance should be employed [36]:

(5) $$T_{p}\left(x,y\right)=\phi_{p}^{*}\left(x,y\right),$$

where the asterisk denotes the complex conjugate. After some operations and taking into account orthogonality between mode field distributions [36]:

(6) $$W_{f}\left(u=0,v=0\right)=\frac{1}{i\lambda f}\overset{N}{\underset{n=1}{\sum }}c_{n}\delta_{\mathrm{pn}}\;=\frac{1}{i\lambda f}c_{p},$$

where $\delta_{\mathrm{pn}}$ is the Kronecker delta. If a CCD detector is placed on axis in the focal plane of L_3_, the use of a filter, as given by Equation (5), allows one to obtain information regarding the p-mode amplitude $|c_{p}|$ at the specific focal plane coordinates (u=0, v=0), the intensity at this point being proportional to the modal weight: $I(0,0)=\;{|W_{f}\left(0,0\right)|}^{2}\propto {|c_{p}|}^{2}$.

A simultaneous procedure based on angular multiplexing can be carried out. It consists of employing a filter whose transmittance is a superposition of different T_p_ filters, associating to each p-mode a different wave vector $\left(k_{x,p}{,k}_{y,p}\right)$, so that the total transmittance function is:

(7) $$T\left(x,y\right)=\overset{N}{\underset{p=1}{\sum }}\phi_{p}^{*}\left(x,y\right){\cdot e}^{i\left({\mathrm{xk}}_{x,p}{+\mathrm{yk}}_{y,p}\right)}.$$

By a convenient choice of these wave vectors, information regarding the different mode modal weights appears at separated-enough points in the focal plane, $u_{p}{=\lambda \mathrm{fk}}_{x,p}$ and $v_{p}{=\lambda \mathrm{fk}}_{y,p}$ being the coordinates of each one of them.

For some applications, it is enough to obtain the modal weights, ${|c_{p}|}^{2}$. However, sometimes it is required to determine the phase difference between modes. According to [36], in order to measure it, it is necessary to use two transmittance functions, defined as

(8) $$T_{p}^{\cos}\left(x,y\right)=\frac{1}{\sqrt{2}}\left[\phi_{0}^{*}\left(x,y\right)+\phi_{p}^{*}\left(x,y\right)\right]{\cdot e}^{i\left({\mathrm{xk}}_{x}{+\mathrm{yk}}_{y}\right)}$$

and

(9) $$T_{p}^{\sin}\left(x,y\right)=\frac{1}{\sqrt{2}}\left[\phi_{0}^{*}\left(x,y\right)+i\phi_{p}^{*}\left(x,y\right)\right]{\cdot e}^{i\left({\mathrm{xk}}_{x}{+\mathrm{yk}}_{y}\right)},$$

so that the p-mode relative phase, $\varphi_{p}$, can be determined with respect to the 0-mode one. In these cases, the intensity at the CCD plane is proportional to the cosine and sine, respectively, of the phase difference [36]. This allows one to obtain an unambiguous solution for the phase differences.

### 2.2. Double-Phase Method for Complex Amplitude Encoding

When implementing the CGH in a phase SLM, all the transmittance functions defined in the previous subsection require the encoding of a complex amplitude in an only-phase device. Among the different proposed procedures for pixel grouping, the double-phase CGHs have been chosen [45] due to their simple implementation and maximum resolution, as in this technique macropixels are composed of only two pixels. This method is based on the principle that any complex value inside the unit circle can be resolved into the sum of two complex values with unit modules.

Consider a CGH implemented in a commercial SLM that only admits phase modulation of its pixels, and a combination of them in macropixels that may offer an approximate complex modulation. Specifically, as explained in [45], a CGH filter with an approximate transmittance to the theoretical one can be achieved by means of grouping pairs of pixels into a macropixel. Considering that each couple of consecutive pixels constitute the macropixel with indexes (m, n), if the desired complex transmittance of the macropixel (m, n) is $T_{\mathrm{mn}}=|T_{\mathrm{mn}}|e^{{i\alpha }_{\mathrm{mn}}}$, the following respective phase shifts are assigned to the two consecutive pixels [45]:

(10) $$\alpha_{\mathrm{mn}}^{\left(1\right)}{=\alpha }_{\mathrm{mn}}{\;-\;\Delta }_{\mathrm{mn}}{\;\mathrm{and}\;\alpha }_{\mathrm{mn}}^{\left(2\right)}{=\alpha }_{\mathrm{mn}}{+\Delta }_{\mathrm{mn}},$$

where $|T_{\mathrm{mn}}|\;\leq \;1$, $0\;\leq {\;\alpha }_{\mathrm{mn}}\;\leq \;2\pi$ and

(11) $$\Delta_{\mathrm{mn}}{=\cos}^{-1}\left(|T_{\mathrm{mn}}|\right).$$

Denoting by Q the transmittance function of the phase-only device where the double-phase CGH are implemented, its Fourier transform can be expressed as:

(12) $$\tilde{Q}={\tilde{Q}}_{S}+{\tilde{Q}}_{N},$$

in such a way that [45]

(13) $${\tilde{Q}}_{S}=ℱ[T(x,y)].$$

Contribution ${\tilde{Q}}_{S}$, referred to as signal, is the only one that should be expected if the double-phase solution provided an exact T(x,y) transmittance. Nevertheless, another contribution to $\tilde{Q}$ also appears, due to the double-phase implementation: ${\tilde{Q}}_{N}$, referred to as noise.

## 3. Modal Analysis Setup

**Figure 2:**
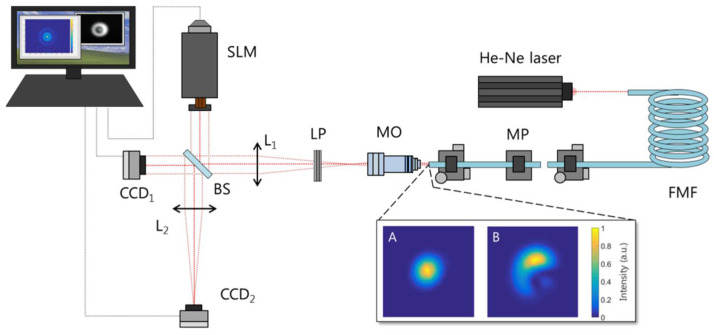
Scheme of the modal analysis setup. FMF: few mode fiber, MP: micro-positioners, MO: microscope objective, L_1_ and L_2_: lenses, LP: linear polarizer, BS: beam splitter, CCD: cameras, SLM: spatial light modulator; A and B are the measured transversal intensity distributions in order to align the setup and test the proposed techniques, respectively.

The required experimental setup is depicted in Figure 2. An SMF28 optical fiber supporting six propagation LP-modes is illuminated by a He-Ne laser (λ=632.8 nm). The first section of optical fiber is repeatedly curved in order to eliminate modes other than the fundamental one, ${\mathrm{LP}}_{01}$. We use this fiber to illuminate a second optical fiber with the same characteristics. This way, when both fibers are placed closely one after another over the same longitudinal axis, the ${\mathrm{LP}}_{01}$ mode is the only one that propagates along the second section of optical fiber. It can be used to carry out the system alignment. By sideways displacing the first fiber with respect to the second one, different transversal distributions are generated, due to different combinations of excited modes, which is useful for testing the MD performance. Figure 2A,B show two examples: the first one with both fiber sections totally aligned, used to calibrate the setup, and the second one at an arbitrary position, employed to test the proposed technique’s performance.

In order to increase the effective resolution, the fiber output beam size has been magnified at the SLM display using a microscope objective (DIN × 40) and a lens (f_1_ = 50 cm) following a 4f configuration (theoretical magnification 125). The 4f-lens combination images the fiber output field distribution through the beam splitter (BS) both in the CCD_1_ camera and in the phase-only SLM operating in reflection mode (PLUTO VIS Phase Only Spatial Light Modulator from Holoeye [49]). As it only operates in the horizontal polarization, we select this state of polarization from the input beam with a linear polarizer (LP). In order to obtain a complete MD, the same procedure should be performed for the vertical polarization. In order to do that, the horizontal LP should be replaced by a vertical one, followed by a half-wave plate. The reflected field from the SLM, where the CGH is implemented, is Fourier transformed by lens L_2_ (f_2_ = 15 cm), and this signal is detected by the CCD_2_ camera, placed at the L_2_ lens focal plane, where the modal weights are obtained by measuring the intensity in the specified coordinates according to Equation (6). In order to avoid interferences, The CCD_2_ and the SLM are covered when measuring intensity profiles with the CCD_1_ camera. The SLM was previously calibrated following the procedure described in [50].

## 4. Correlation-Filter Size adjustment

**Figure 3:**
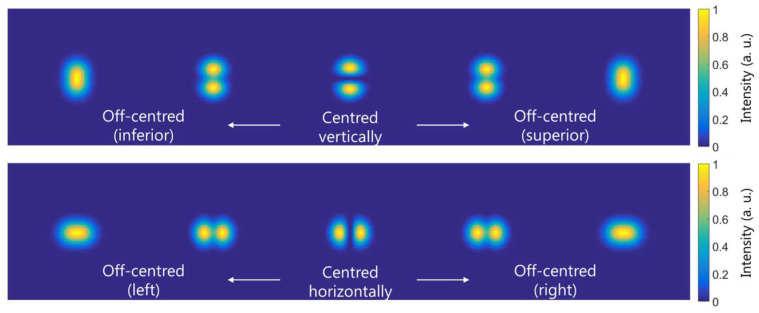
Simulated transversal intensity distributions at the CCD_2_ plane, when the CGH is illuminated with the ${\mathrm{LP}}_{01}^{\;}$ mode and the (**a**) ${\mathrm{LP}}_{11}^{e}$ and (**b**) ${\mathrm{LP}}_{11}^{e}$ modes are implemented in the SLM, for different transversal displacements of the CGH. The optical axis point is marked with a red cross.

The MD performance is based on the overlap in the SLM between the incident light beam and the implemented CGH. Inaccuracies in the position or size of the encoded CGH with respect to the incident light can result in the detection of erroneous modes or in the incorrect determination of the modal weights. Notwithstanding its importance, these issues have not been studied in depth, except for in a recently published tutorial [41]. Their proposed approach to transversally align the CGH position consists of displacing the filter position in such a way that, for a mismatched overlap, the on-axis null appears centered regarding the input light beam. If the first and second optical fiber sections are aligned so as to achieve single ${\mathrm{LP}}_{01}$ mode propagation in the second one, there should be zero on-axis intensity when implementing any other mode into the match filter. We can benefit from ${\mathrm{LP}}_{11}^{e}$ and ${\mathrm{LP}}_{11}^{o}$ mode symmetry in order to center the CGH vertically and horizontally, respectively. The simulated transversal intensity distributions at the CCD_2_ plane, when the ${\mathrm{LP}}_{11}^{e}$ and ${\mathrm{LP}}_{11}^{e}$ modes are implemented in the SLM, are shown in Figure 3a,b, respectively, for different cases of transversal centering. As one can see, when the filters are centered with respect to the incident field, there is zero intensity in the optical axis point (marked with a red cross). As the filters decenter, the intensity at the measurement position increases.

**Figure 4:**
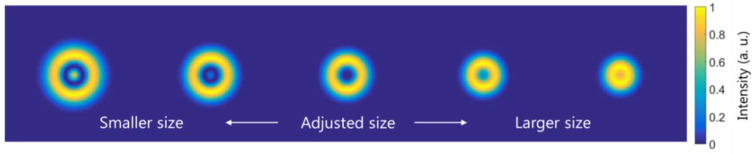
Simulated transversal intensity distributions at the L_2_ back focal plane when the CGH is illuminated with the ${\mathrm{LP}}_{01}^{\;}$ mode and the ${\mathrm{LP}}_{02}^{\;}$ mode is implemented in the SLM for different magnification values of the CGH.

We propose that this same idea can be used to adjust the filter size. Despite it being straightforward to calculate the theoretical 4f-magnification, this value can be slightly modified in the experimental implementation, mainly because it is not easy to place the optical fiber output end at the exact focal length distance from the microscope principal plane. In Ref. [51], a method for filter-size adjustment is proposed, for which it is required to obtain some parameters, such as the beam quality factor and the second moment. Here, we present a different approach, based on the ${\mathrm{LP}}_{02}^{\;}$ mode symmetry. If we implement the ${\mathrm{LP}}_{02}$ mode into the CGH and illuminate it with the ${\mathrm{LP}}_{01}$ mode, only when both their sizes are matched will there be zero intensity at the optical axis, as shown in the simulations from Figure 4.

**Figure 5:**
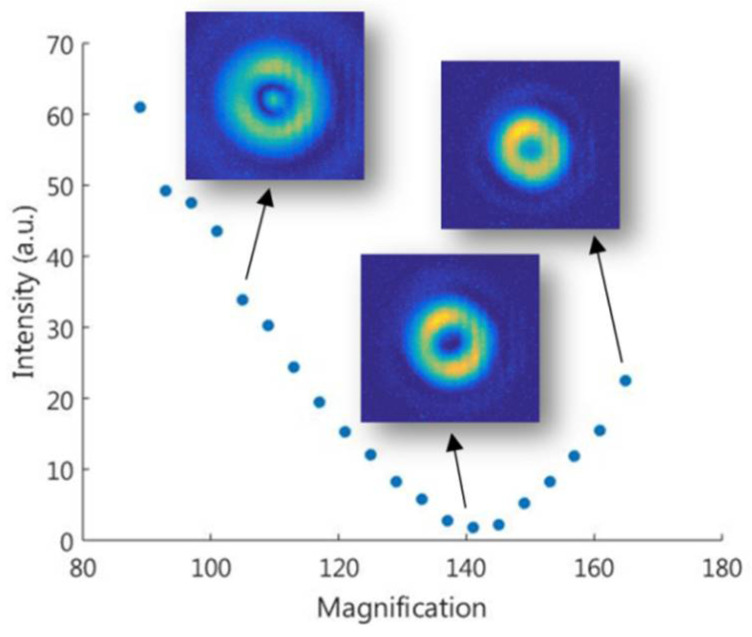
Measured intensity at the CCD_2_ camera optical axis point as a function of the ${\mathrm{LP}}_{02}^{\;}$ mode magnification implemented in the SLM when illuminated with the ${\mathrm{LP}}_{01}^{\;}$ mode. Three measured transversal intensity distributions are superimposed for three specified points.

We can use this behavior to find the correct scale just by minimizing the optical axis intensity as a function of the theoretical magnification value. This is shown experimentally in Figure 5. Overlaying the graph, three measured transversal intensity distributions are shown for a smaller, adjusted, and larger size (from left to right) of the filter with respect to the incident light. The measured intensity distributions follow a similar behavior than the simulated ones in Figure 4.

According to the minimum intensity, the magnification value is 141, which has a 13% relative error with respect to the theoretical one. This error can be explained due to the inherent uncertainties of the optical system positioning. This method can be used as long as the set of modes has the required symmetry, which is common in cylindrical-like structures.

## 5. Double Phase Method Noise Term Analysis

Consider a double-phase CGH with transmittance Q(x,y) (Equation (18)), implemented in order to emulate an ideal CGH filter with complex transmittance T(x,y) (Equation (13)). According to Equations (3), (4), and (12), this double-phase filter yields a field distribution in the L_2_ back focal plane, such as:

(14) $$W_{f}=\frac{1}{i\lambda f}[\tilde{U}*({\tilde{Q}}_{S}+{\tilde{Q}}_{N})],$$

where $\tilde{U}=ℱ\left[U\left(x,y\right)\right]$ and the (∗) sign represents convolution product. We consider two different contributions to $W_{f}$, called here signal and noise terms, respectively:

(15) $$W_{f,S}=\frac{1}{i\lambda f}[\tilde{U}*{\tilde{Q}}_{S}],$$

(16) $$W_{f,N}=\frac{1}{i\lambda f}[\tilde{U}*{\tilde{Q}}_{N}],$$

Because ${\tilde{Q}}_{S}=ℱ\left[T\left(x,y\right)\right]$, $W_{f,S}$ represents the field distribution that would be obtained if an ideal CGH filter with complex transmittance T(x,y) was employed. Nevertheless, a superimposed noise term $W_{f,N}$ is also present. As stated in Equation (16), this term contains the convolution of two factors. One of them, ${\tilde{Q}}_{N}$, is intrinsic to the filter, while the other, $\tilde{U}$, is directly related to the input electric field distribution, which is obviously unpredictable as it is the object of analysis. Therefore, the magnitude and distribution of the noise term is expected to be very different depending on each working condition. For this reason, its impact cannot be analyzed by means of a general mathematical treatment.

**Figure 6:**
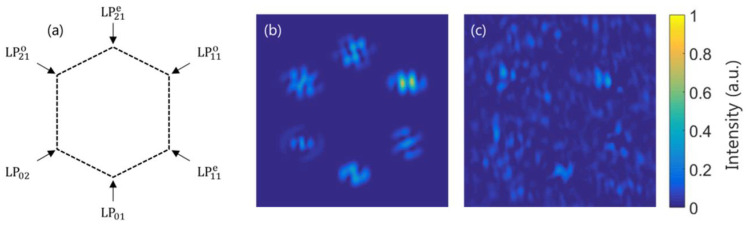
(**a**) Simultaneous MD spatial configuration scheme; (**b**,**c**) normalized transversal intensity distributions $W_{f,S}$ and $W_{f,N}$, respectively, when all six LP-modes are equally present in the incident light.

A case in which the noise factor strongly affects the MD is shown in Figure 6. Suppose an incident beam with all six LP-modes equally present and a CGH in which all the match filters (one for each mode) are simultaneously multiplexed with a different grating to spatially separate the signals at the Fourier plane. Specifically, the vertexes of a regular hexagon, as shown in Figure 6a–c, show the simulated intensity at the L_2_ back focal plane, separated into the signal term $W_{f,S}$ (Equation (15)) and the noise term $W_{f,N}$ (Equation (16)), respectively.

By comparing the modal weight percentages with and without noise terms, relative errors up to 50% are obtained, as shown in Table 1, thus preventing the correct performance of the MD. The modal weight in percentage and the relative error are computed by ${|c_{l}|}^{2}(\%)=100{|c_{l}|}^{2}/\overset{N}{\underset{n=1}{\sum }}{|c_{n}|}^{2}$ and $\varepsilon_{r}=100\left({|c|}_{\mathrm{Exp}}^{2}-{|c|}_{\mathrm{Teo}}^{2}\right)/{|c|}_{\mathrm{Teo}}^{2}$, respectively, where ${|c|}_{\mathrm{Exp}}^{2}$ and ${|c|}_{\mathrm{Teo}}^{2}$ are the experimental and theoretical modal weights, the last one being 16.67% as all modes are equally present.

**Figure 7:**
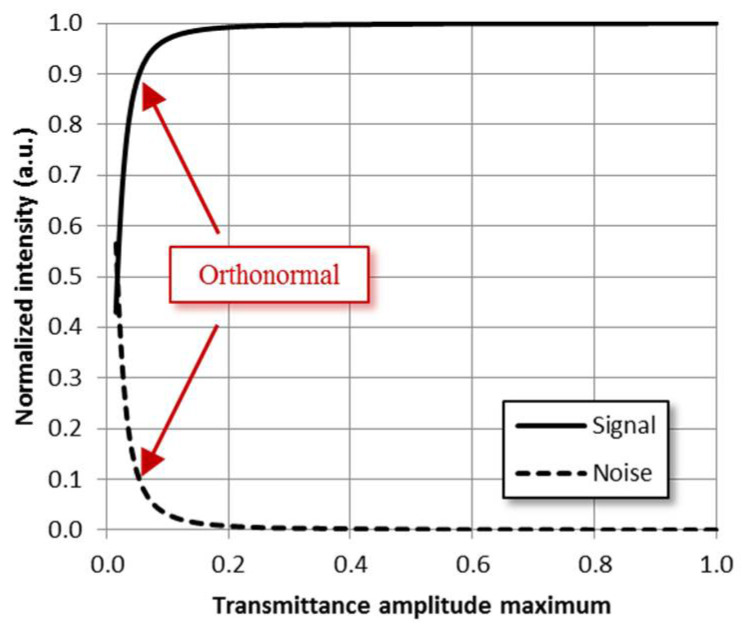
Signal and noise intensity evolution as a function of the maximum value in the transmittance function (Equation (7)) for the configuration case shown in Figure 6.

In view of the findings, it is essential to reduce the noise term influence on the MD performance. One way to achieve this is to realize the MD sequentially rather than simultaneously. However, it is not possible to obtain all modal weights in real time with this procedure, which can be an important disadvantage depending on the system dynamics. Another approach is to normalize the transmittance function (Equation (7)). Theoretically, any transmittance value between zero and one is valid when implementing the double phase method. Nevertheless, in order to use the whole range of the SLM and reduce the noise impact, the maximum value of the transmittance function amplitude should be as close as possible to one. This affects parameter $\Delta_{\mathrm{mn}}$ from Equation (11), which is necessary for the implementation of the double phase method. The normalization has a direct impact on the signal to noise ratio, as shown in Figure 7, where we have used the same configuration than the one in Figure 6 and summed the intensities at the six information positions for both the signal (Equation (15)) and the noise (Equation (16)) terms.

As shown in Figure 7, by adjusting the maximum value of the transmittance amplitude to one, we improve the signal to noise ratio, while if we keep the set of modes orthonormality (situation marked with the red arrows), the noise intensity cannot be neglected with respect to the signal, thus affecting the MD. When all the modes are measured using the same transmittance function (simultaneous MD case), the renormalization appears as a constant factor equal for all measured modal weights. However, the renormalization factors must be considered when measuring different modal weights with different transmittance functions in order not to break the mode orthonormality.

## 6. Robustness Improvement in Relative Phase Measurements

In theory, the phase difference between modes can be unambiguously determined from two measurements by using Equations (8) and (9), each one having two possible solutions. Nevertheless, the mode relative phase measurements can be easily influenced by system instabilities (i.e., laser power fluctuations, micro-positioners looseness, etc.) or by misalignments due to position uncertainties (i.e., lenses, fiber, CCD, SLM). As a consequence, there may be an error in the arccosine and arcsine result that could prevent correct phase retrieval. For such cases, we propose a different transmittance function in order to measure the relative phases and also to study the phase determination uncertainty.

Suppose we implement in the SLM the following transmittance function:

(17) $$T\left(x,y\right)=\frac{1}{\sqrt{2}}\left[\phi_{0}^{*}\left(x,y\right)+\phi_{l}^{*}\left(x,y\right){\cdot e}^{i\theta }\right],$$

as a function of a variable phase θ. The particular cases θ=0 and θ=π/2 recover Equations (8) and (9), respectively. The intensity at the CCD_2_ measurement position as a function of θ is

(18) $${|W_{f}^{\;}\left({u=\lambda \mathrm{fk}}_{x}{,v=\lambda \mathrm{fk}}_{y},\theta \right)|}^{2}\propto {|c_{0}|}^{2}+{|c_{l}|}^{2}+2|c_{0}||c_{l}|{\cdot \cos(\varphi }_{l}+\theta ).$$

In order to improve the robustness of the relative phase determination, we perform a series of intensity measurements as a function of θ and fit them to the function in Equation (18).

**Figure 8:**
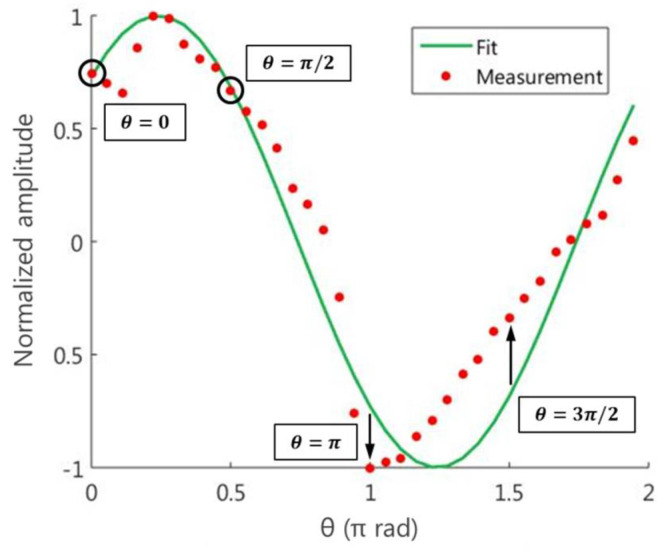
Relative phase measurements of the ${\mathrm{LP}}_{21}^{e}$ mode together with its cosine least-squares best fit, based on Equation (18), for the transversal intensity distribution shown in Figure 2b.

As an example, Figure 8 shows the so mentioned measurements of the phase difference between the ${\mathrm{LP}}_{21}^{e}$ and ${\mathrm{LP}}_{01}^{\;}$ modes, when the input distribution is the one shown in Figure 2b, together with its best fit by least squares. It is clear to see that, despite the fact that the experimental dots follow a similar behavior than the cosine function, there are some significant differences with respect to the fitting function. Both instabilities (i.e., laser fluctuations, fiber disturbances) and experimental inaccuracies (i.e., devices alignments) may be responsible for the lack of agreement between the measurements and the theoretical function.

Superimposed to the graph in Figure 8, four points have been highlighted. First, two points, θ=0 and θ=π/2, are the particular cases from Equations (8) and (9). In this case, the points show a good agreement with the fitting curve. In fact, following the two-measurements procedure, a phase difference of 0.23π rad is obtained, which is almost the same value as the one determined through the fitting approach: 0.24π rad. Based on this result, one could think that the fitting approach does not improve the phase determination. However, the relative phase obtained through the two-measurements procedure may depend heavily on the selected points, as one can see in Figure 8. Suppose we choose the particular cases θ=π and θ=3π/2, whose experimental values are far from the fitting curve, and compute the phase difference. In this case, the obtained angles do not match each other. The obtained phases that fall in the same quadrant are 0 and 0.39π rad, respectively. It is clear to see that, in general, the function fit allows us to obtain a relative phase less influenced by possible instabilities and uncertainties. In the next section, we observe its impact by performing a wavefront reconstruction.

## 7. Wavefront Reconstruction

**Figure 9:**
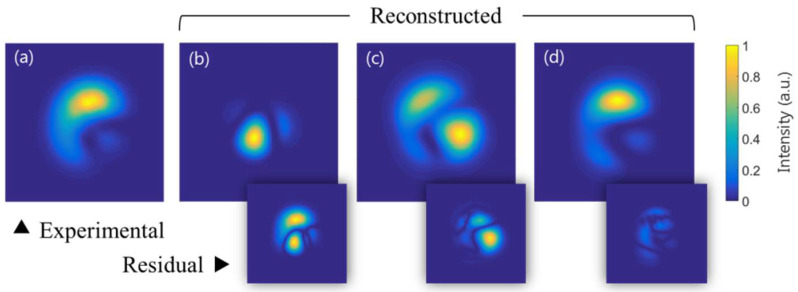
Arbitrary measured transversal intensity distribution (**a**) together with their numerical reconstructions (**b**) when the size is not readjusted through the proposed technique in Section 4, (**c**) when the phases are determined without using the fitting procedure presented in Section 6, and (**d**) when both proposed techniques are employed. The residual intensity patterns are shown below each reconstructed image.

In order to test the proposed size adjustment technique and phase retrieval procedure, together with the MD method itself, we have reconstructed the transversal intensity distribution shown in Figure 2b. Figure 9 shows the comparison between the experimental distribution (a) and the numerically reconstructed ones (b–d). The reconstructed distributions have been obtained by measuring all six LP-mode weights and relative phases. However, in the reconstructions shown in Figure 9b,c, the proposed CGH size adjustment and phase retrieval techniques have not been used, respectively. The reconstruction shown in Figure 9b has been obtained by employing the proposed phase retrieval fitting approach but not the CGH size adjustment. Instead, its size has been computed theoretically with the 4f magnification. On the contrary, for the reconstruction shown in Figure 9c, the CGH size adjustment technique has been used, but the relative phases have been determined through the two-measurements method. Finally, the distribution shown in Figure 9d has been computed using both of the proposed techniques. As one can see, a good agreement is shown between Figure 9a,d distributions, highlighting the importance of both the size adjustment and the phase retrieval. The residual intensity patterns, computed where m and r mean measured and reconstructed, are respectively provided, together with each reconstructed image.

By comparing the transversal intensity distributions one can see that the CGH size adjustment is quite critical and that, although a look alike distribution is obtained without determining the relative phases with our proposed procedure, a better reconstruction is obtained when employed. To quantitatively compare the intensity distributions from Figure 9, the root-mean-square error (RMSE) has been computed for each of the reconstructed images with respect to the experimentally registered one, all four of them previously normalized to the unit, obtaining the RMSE values 2.4 × 10^−2^, 1.8 × 10^−2^, and 1.7 × 10^−3^ for the Figure 9b–d intensity distributions, respectively. Distribution from Figure 9d gives a RMSE one order of magnitude lower than the ones from Figure 9b,c, showing the good performance of the reconstruction when using the proposed procedures. Table 2 summarizes the modal weights and relative phases obtained by using the proposed techniques, with which distribution from Figure 9d is computed.

## 8. Conclusions

We have presented a series of practical procedures to perform an accurate modal decomposition of light, and we tested them satisfactorily by wavefront reconstruction in a few-mode fiber, allowing us to verify both the good performance of the modal analysis procedure and the proposed techniques. In order to reduce the SNR when performing a simultaneous MD and exploit the SLM range, the set of modes orthonormality needs to be broken, and thus a scale factor taken into account. It has been shown that it is possible to benefit from the LP-mode symmetry, not only to transversally center the CGH, but also to adjust its size with respect to the incident beam. This procedure is not restricted to the LP-modes, and it can be used as long as the set of modes provides the necessary symmetry, which is usually fulfilled in cylindrical-like waveguides. When setup instabilities make the correct phase difference determination through the classical approach difficult, a possibility to improve phase retrieval robustness consists of implementing a transmittance as a function of an angle parameter and adjust the set of measurements. The good performance of these techniques has been successfully tested by wavefront reconstruction.

## Figures and Tables

**Table 1:** Simulated modal weights (obtained with the $W_{f}$ function) and relative errors (obtained by comparing them with the ones determined through the $W_{f,S}$ function) following the simultaneous configuration of Figure 6 when all six LP-modes are equally present in the incident light.

Mode	Weight (%)	Relative Error (%)
${\mathrm{LP}}_{01}^{\;}$	23.4	40
${\mathrm{LP}}_{11}^{e}$	14.1	−15
${\mathrm{LP}}_{11}^{o}$	7.8	−53
${\mathrm{LP}}_{21}^{e}$	17.2	3
${\mathrm{LP}}_{21}^{o}$	17.2	3
${\mathrm{LP}}_{02}^{\;}$	20.3	22

**Table 2:** Measured modal weights and relative phases to that of the LP_01_ mode from the output intensity distribution shown in Figure 9a.

Mode	Weight (%)	Phase (π rad)
${\mathrm{LP}}_{01}^{\;}$	33	0.0
${\mathrm{LP}}_{11}^{e}$	25	1.0
${\mathrm{LP}}_{11}^{o}$	10	1.9
${\mathrm{LP}}_{11}^{e}$	14	0.2
${\mathrm{LP}}_{11}^{o}$	3	0.9
${\mathrm{LP}}_{02}^{\;}$	16	0.9

## Data Availability

Data available on request.
